# Birt-Hogg-Dubé syndrome with simultaneous hyperplastic polyposis of the gastrointestinal tract: case report and review of the literature

**DOI:** 10.1186/s12881-020-0991-8

**Published:** 2020-03-14

**Authors:** Flávia Balsamo, Pedro Augusto Soffner Cardoso, Sergio Aparecido do Amaral Junior, Therésè Rachell Theodoro, Flavia de Sousa Gehrke, Maria Aparecida da Silva Pinhal, Bianca Bianco, Jaques Waisberg

**Affiliations:** 1 Section of General Surgery and Gastrointestinal Surgery, Department of Surgery I, Faculdade de Medicina do ABC, Avenida Lauro Gomes, 2000, Santo André/São Paulo, CEP 09060-870 Brazil; 2 Section of Biochemistry, Department of Morfology and Physiology, Faculdade de Medicina do ABC, Avenida Lauro Gomes, 2000, Santo André/São Paulo, CEP 09060-870 Brazil; 3 Section of Sexual and Reproductive Health and Populational Genetics, Department of Collective Health, Faculdade de Medicina do ABC, Avenida Lauro Gomes, 2000, Santo André/São Paulo, CEP 09060-870 Brazil

**Keywords:** Birt-Hogg-Dubé syndrome, FLCN gene, Skin neoplasms, Pneumothorax, Polyps

## Abstract

**Background:**

Birt-Hogg-Dubé syndrome (BHDS) is a rare autosomal dominant genodermatosis characterized by benign growth of the hair follicles, the presence of pulmonary cysts, spontaneous pneumothorax, and bilateral renal tumors that are usually hybrid oncocytic or multifocal chromophobe renal cell carcinoma. The diagnosis is confirmed by the presence of a pathogenic variant in the tumor suppressor folliculin (*FLCN*) gene mapped at 17p11.2. Although the dermatological lesions typical of BHDS are benign and only cause aesthetic concerns, and the pulmonary manifestations are controllable, the greater tendency of patients with this syndrome to present benign or malignant renal tumors, often bilateral and multifocal, makes the diagnosis of this syndrome important for the prognosis of the patients. The objective was to report the case of a patient with BHDS, without pulmonary manifestations and with hyperplastic polyposis of the gastrointestinal tract, and to perform a literature review.

**Case presentation:**

A 60-year-old man complained of abdominal pain and diarrhoea for 2 months. Physical examination was normal except for the presence of normochromic papules in the frontal region of the face associated with hyperkeratotic and hyperchromic papules in the dorsal region. The excisional biopsies of the skin lesions indicated trichodiscomas. Esophagogastroduodenoscopy, enteroscopy, and colonoscopy showed the presence of hyperplastic polyps in the stomach, duodenum, jejunum, colon, and rectum. Computed tomography (CT) and magnetic resonance imaging (MRI) of the abdomen revealed multiple expansive solid lesions in both kidneys, with necrotic and calcified areas. Renal magnetic resonance angiography also showed a solid lesion in the right kidney measuring 5 cm in diameter and another solid lesion in the left kidney measuring 8 cm in diameter, both suggestive of renal angiomyolipoma. CT scans of the skull, chest, and temporal bones were normal. The genetic study revealed the presence of a variant of *FLCN i*n the intron 13.

**Conclusions:**

To the best of our knowledge, this is the first reported case of BHDS with the simultaneous finding of gastrointestinal hyperplastic polyposis, which may represent a possible phenotypic expression of this syndrome that has not yet been described.

## Background

Birt-Hogg-Dubé syndrome (BHDS) (MIM 135150) or Hornstein-Knickenberg syndrome is an autosomal-dominant hereditary disorder associated with the germline pathogenic variant of the folliculin gene (*FLCN*), which causes a predisposition for the risk of benign cutaneous fibrofolliculomas, pulmonary cysts, spontaneous pneumothorax, and multiple, mainly malignant, bilateral and multifocal renal neoplasias [1, 2]. BHDS is a rare syndrome that occurs in 1/200,000 people [2–4].

The majority of patients with BHDS present dermatological manifestations and 90% are cutaneous fibrofolliculomas. Individuals with BHDS are 32 to 50 times more likely to develop spontaneous pneumothorax than the unaffected population [3–5], and 30% report a history of spontaneous pneumothorax, usually before age 40 [4]. Renal tumors occur in 25 to 35% of the cases, and the most frequent type of neoplasm is the hybrid or chromophobe oncocytic tumor, which exhibits aspects of chromophobe carcinoma and oncocytoma in the same tumour and represents half of the renal neoplasms found in patients with BHDS. The risk of developing renal tumors is seven times higher in individuals with BHDS than in non-affected siblings [6, 7].

*FLCN*, the only known gene related to BHDS, is considered a tumor suppressor, mapped at 17p11.2, and it contains 14 exons, 11 of which encode the protein folliculin (FLCN) [8]. BHDS can also occur as a function of a pathogenic de novo germline *FLCN* variant in an individual with no previous family history [4, 6, 8]. *FLCN* is expressed in normal skin cells, nephrons, stromal cells, type I pneumocytes, and acinar cells of the pancreas and parotid gland. Pathogenic *FLCN* variants may interfere with the ability of FLCN to restrict cell growth and division, leading to the formation of malignant and benign tumors [6–8]. FLCN is associated with AMP-activated protein kinase (AMPK), hypoxia-inducible factors (HIF), transforming growth factor beta (TGF-β), and Mammalian target of rapamycin (mTOR) -signalling pathways, its inactivation determines the increase in the mitochondrial oxidative metabolism, and pathogenic variants in these signaling pathways result in deregulated cell growth and protein synthesis [3–6, 9]. The participation of *FLCN* in the mTOR pathway may explain the phenotype similarity between BHDS and hereditary hamartomatous syndromes such as Cowden’s syndrome, tuberous sclerosis, and Peutz-Jeghers syndrome [5, 6].

*FLCN* is a tumor suppressor gene that fits the two-hit model for tumor suppression [6]. Individuals with BHDS are born with a variant copy of *FLCN* in each cell and somatic mutations and/or loss of heterozygosity are required in the second copy of the gene for renal carcinogenesis [10]. Random pathogenic variants may inactivate the normal copy of the *FLCN* gene in a subset of cells, and when this occurs, the result is that these cells do not have functional copies of *FLCN*, allowing them to grow uncontrolled [3, 6–8]. This loss of heterozygosity is frequently detected in renal tumors associated with BHDS and is considered necessary for the appearance of renal tumors [6–9]. On the other hand, loss of heterozygosity was not evident in BHD-associated fibrofolliculomas. Previous histopathologic analyses suggested that *FLCN* probably exists in a haploinsufficient form without somatic second hit mutations in the cyst-lining cells [5].

Germline pathogenic variants in *FLCN* are found in 84 to 88% of families with BHDS. No correlation was identified between the pathogenic variant type or location within *FLCN* gene and any of the phenotypic manifestations [3, 6], except the pathogenic variants of BHDS in exon 9, which were associated with a greater number of pulmonary cysts than the pathogenic variants in other exons [3, 5].

The *FLCN* variant patterns consist predominantly of deletions, insertions, missense, nonsense, and splice sites [6, 7], which result in premature protein truncation and a presumed loss of gene function [3, 6, 8]. In addition, intragenic deletions and duplications that alter the protein structure [6] have also been reported. The *FLCN* gene with 14 exons, of which 11 are coding exons, encode a 579 amino acid protein, folliculin, which is highly conserved among species. A total of 149 unique germline pathogenic variants of *FLCN* were identified and cataloged in the Leiden Open Variation Database [11], and there are no sequence variants reported in the first 3 non-coding regions, only intragenic deletions that delete exon 1, the putative site for the *FLCN* promoter, although no phenotype-genotype correlation has been found so far. Germline insertion or deletion of a cytosine in an eight-cytosine (C8) mononucleotide (c.1285dupC or c.1285delC) in exon 11 and the deletion of a guanine at nucleotide 454 (c. 454delG) in exon 4, which interferes in the *FLCN* initiator codon, represent the most 5′ and most 3′ coding sequence mutations reported in the literature [3, 6, 7].

The following health conditions have been reported in at least one BHDS patient: prostatic carcinoma, breast sarcoma, jaw carcinoma, colorectal carcinoma, lipomas, parathyroid adenomas, parotid oncocytomas, thyroid cancer, colonic polyposis, progressive chorioretinopathy and chorioretinal scarring, breast fibroadenomatosis, parotid adenomas, neural tissue tumors, focal cutaneous mucinosis, cutaneous leiomyoma, internal carotid artery aplasia, basal cell and squamous cell carcinoma, and dermatofibrosarcoma protuberans has been described [2–4, 6, 8, 9].

Association of polyps in the large intestine with BHDS is rare [3, 6, 8]. Microscopically, hyperplastic polyps are defined by luminal and crypt serration with normal architecture and normal proliferative characteristics [12, 13]. In the large intestine, these tumors are frequently detected in adults and the elderly [14] and may be solitary or multiple. They are usually smaller than 5 mm in diameter, are more frequent in the distal colon and rectum, and are usually asymptomatic. Most colorectal hyperplastic polyps have no malignant potential, but some may progress to colorectal carcinoma [14].

Gastric hyperplastic polyps are the most common type of polyps found in the stomach, typically occurring in a scenario of mucosal injury, most often chronic gastritis associated with *Helicobacter pylori* infection and autoimmune gastritis. Although preferentially located in the gastric antrum, hyperplastic polyps can occur anywhere in the stomach, including the body, fundus, and cardia, with or without antrum involvement. Hyperplastic gastric polyps are usually single, small, and sessile but may be pedunculated or multiple. There is no predominance in men or women; they may be asymptomatic and may be an incidental finding in gastroscopy [15]. They are considered benign lesions, but a minority of cases may progress to dysplasia [0.2 to 10%] or adenocarcinoma [0.6 to 3%] [16].

Hyperplastic polyps in the duodenum are very rare. We found only three previous reports of hyperplastic polyps in the duodenum in a total of 12 described cases [12, 17, 18]. We did not find reports in the literature regarding the presence of hyperplastic polyps in the jejunum.

The objective of this study was to describe the case of a patient with BHDS associated with the simultaneous involvement of the stomach, duodenum, proximal jejunum, and colon with hyperplastic polyps, and to perform a literature review.

## Case presentation

The case report followed CARE guidelines.

**Fig. 1:**
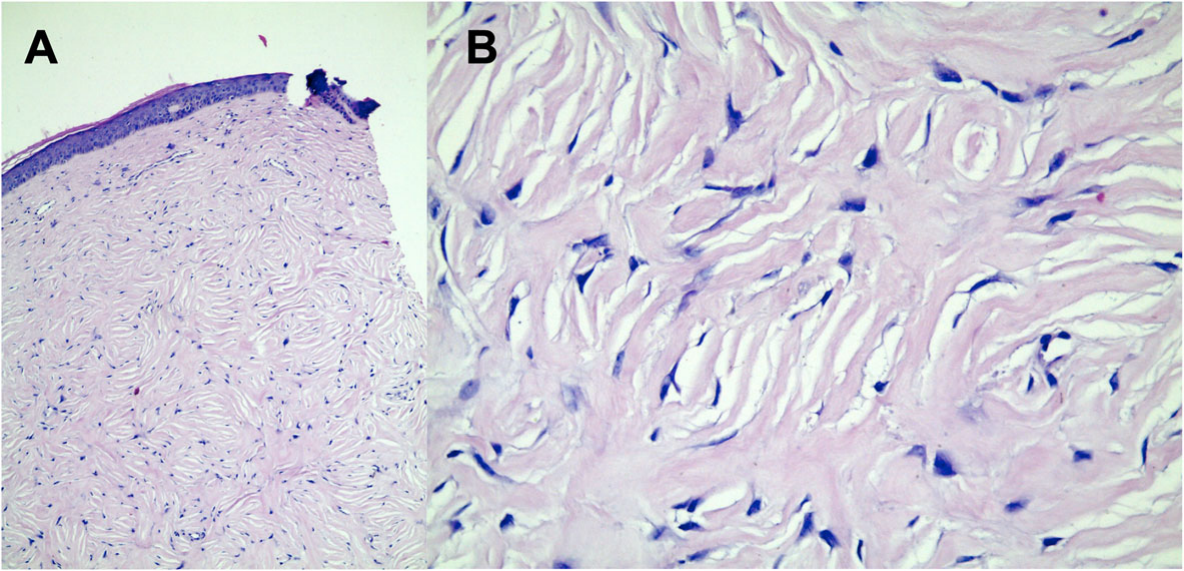
Fibroepithelial polyp (skin fibroma). **a** Histological skin sections exhibit a markedly rectified epidermis. In the dermis, mesenchymal cellular proliferation can be observed, consisting of spindle cells with the cytoplasm sometimes indistinct, sometimes spiculated in appearance, containing spindle nuclei without atypia (HE 200x). **b** The cells are arranged so that “swirls” are sometimes formed among the dense collagenized stroma (HE 400x)

**Fig. 2:**
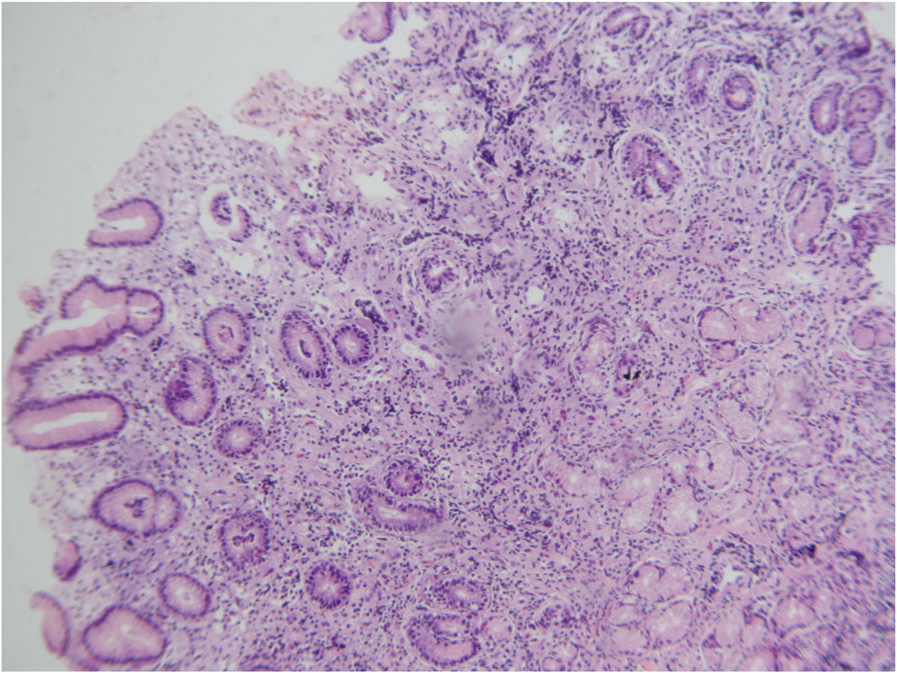
Photomicrograph of a hyperplastic polyp of the gastric mucosa showing foveolar hyperplasia, without cytoarchitectural atypia (HE 200x)

A 60-year-old man, from Santo André/São Paulo, Brazil, complained of abdominal pain and diarrhoea for 2 months. The patient was submitted to the removal of vocal cord angioma, left parotidectomy due to oncocytoma and lipomatosis, and a total thyroidectomy due to carcinoma. The patient had hypothyroidism and hypertension controlled with medication and adult diabetes controlled with oral hypoglycaemic agents and diet. The physical examination was normal except for the presence of normochromic papules in the frontal region with progressive enlargement, associated with brownish hyperkeratotic and hyperchromic papules in the dorsal region, in addition to an erythamatous nodule with telangiectasias in the right upper lip and an erythaematous papule in the nose. All skin lesions, although asymptomatic, were surgically removed. An anatomopathological examination of the dorsal and frontal lesions revealed trichodiscomas (Figs. 1 and 2), and the lesions located in the upper lip and nose were nodular and micronodular solid basal cell carcinomas.

During the diagnostic investigation, colonoscopy identified numerous sessile and pedunculated polypoid lesions located from the sigmoid to the cecum, along the entire colonic mucosa, measuring between 3 and 8 mm, some of which were resected. A histopathological examination of the lesions removed from the ascending, transverse, and sigmoid colon showed hyperplastic polyps of the colonic mucosa (Fig. 1). In the upper digestive endoscopy, a large number of sessile polyps were found in the fundus, body, antrum, and first part of the duodenum, and some were removed. Histopathological examination revealed the presence of gastric hyperplastic polyps (Fig. 2). The *Helicobacter pylori* survey performed by gastric biopsy was positive. The barium study of the small intestine was normal. Enteroscopy showed the presence of large numbers of sessile, subpedunculated, and pedunculated polyps, measuring 3 to 15 mm, in the distal part of the duodenum and the proximal jejunum. The anatomopathological examination of these duodenal and jejunal polypoid lesions also revealed hyperplastic polyps. A capsule endoscopy procedure was not performed.

**Fig. 3:**
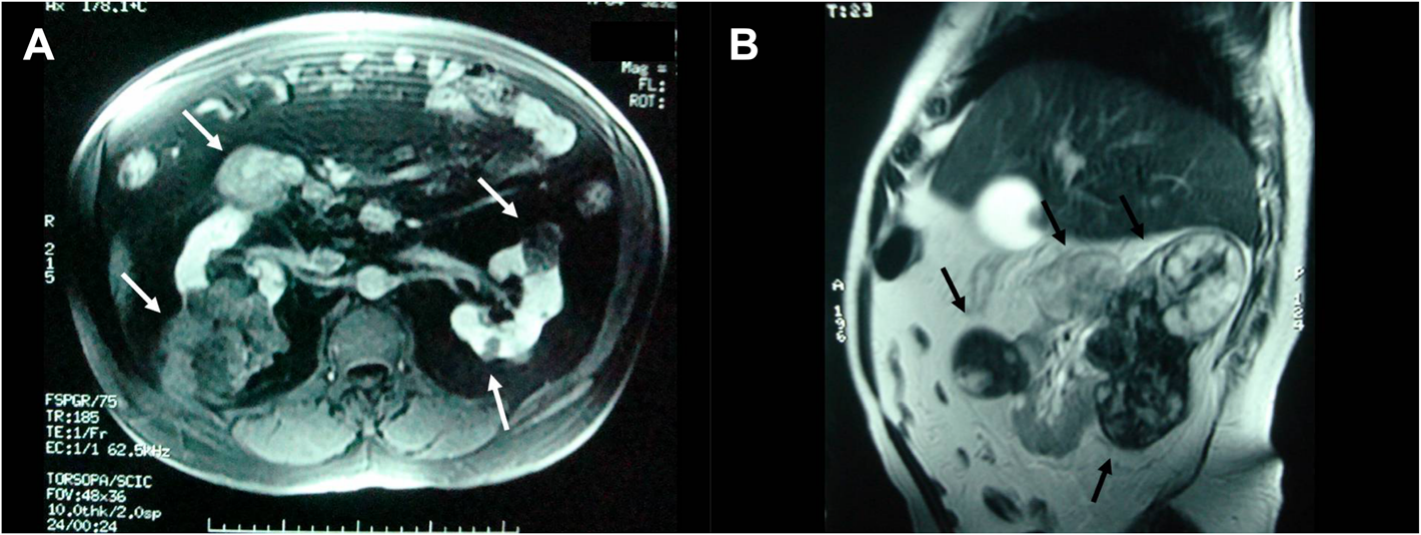
Magnetic resonance imaging (MRI) of the abdomen. **a** T1-weighted image with fat suppression after venous administration of the contrast medium. Several expansive formations can be observed in both kidneys with less intense enhancement than that of the adjacent renal parenchyma (white arrows). **b** T2-weighted image of the sagittal section of the right kidney. The black arrows show multiple expansive heterogeneous signal formations, with hyposignal and hypersignal areas in T2-weighted sequences and a 10-cm lesion in the lower pole of the right kidney

Abdominal ultrasound revealed cholecystolithiasis, a cyst and calcification in the left kidney, and a right retroperitoneal solid mass. Computed tomography (CT) of the abdomen showed the same ultrasound findings, in addition to a 5-cm heterogeneous solid mass located in the right kidney, suggestive of the primary neoplastic process, and cystic lesions in both kidneys. Abdominal magnetic resonance imaging (MRI) with contrast enhancement showed multiple solid expansive lesions in both kidneys, with necrotic areas and punctuate calcifications, predominantly located in the lower two-thirds of both kidneys. The largest lesion was located in the right kidney and was 10 cm in diameter, followed by another one in the left kidney measuring 5 cm (Fig. 3). A renal magnetic resonance angiography showed that both renal masses presented an aspect suggestive of renal angiomyolipoma. The results from the CT of the head, chest, and temporal bones, as well as the MRI of the skull, were normal.

Shortly after undergoing these examinations, the patient had a stroke due to high blood pressure that was confirmed by a cranial CT scan. He developed pneumonia that progressed to septic shock and death despite treatment. An autopsy was not authorized.

In the genetic study, genomic DNA was extracted from the peripheral blood sample using the Illustra Blood GenomicPrep Mini Spin Kit (GE Healthcare, Marlborough, MA, USA) following the manufacturer’s instructions. Genetic variants were named according to the recommendations of the Human Genome Variation Society (HGVS) [19]. The NG_008001.2 of *FLCN* was used as a reference. The primer oligonucleotides used for the amplification of 10 *FLCN* exons was obtained according to Nahorski et al. [20].

Two microliters of the genomic DNA were used for the amplification of the sequence of interest by Polymerase Chain Reaction (PCR), using each primer oligonucleotide pair (10 μM), 10X concentrated buffer containing 20 mM MgSO_4_ (5 μL), and 1.25 units of the Pfu enzyme DNA polymerase, for a total reaction volume of 50 μL. The PCR conditions were 35 cycles of 94 °C for 5 min, 35 cycles of 94 °C for 1 min, 35 cycles of 58 °C for 1 min, and 35 cycles of 72 °C for 1 min. The PCR product was separated by electrophoresis on 2% agarose gel.

The PCR product was purified with the QIAquick Gel Extraction Kit (Qiagen, Valencia, CA, USA), following the manufacturer’s instructions, and eluted in 40 μL of deionized water. The labeled terminators of the ABI Prism BigDye Terminator Ready Reaction Cycle Sequencing Kit (Affimetrix, Santa Clara, CA, USA) and the oligonucleotides were used in the purified fragment, and the automatic sequencing was performed on an automated DNA analyzer (ABI 3130 Genetic Analyzer, Applied Biosystems, Carlsbad, CA, USA). The DNA sequences of the exons were analyzed using the software FinchTV (Geospiza, Seattle, WA, USA) and the BLAST (Basic Local Alignment Search Tool) program (Gene Codes Corporation, Ann Arbor, MI, USA). The search for variants in the *FLCN* gene was performed in ClinVar, Ensemble, UniProt and Mutation Taster.

**Fig. 4:**
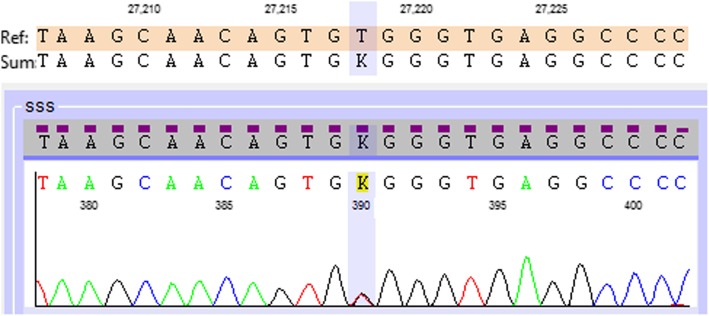
The schematic diagram shows the variant involving *FLC*N intron 13 corresponding to an exchange of timine (T) for guanine (G) at nucleotide 27,218 (NG_008001.2:g.27218 T > G)

A variant was found in heterozygosis that corresponds to a single base exchange of timine (T) for guanine (G) at nucleotide 27,218 (NG_008001.2:g.27218 T > G, NM_144997.7(FLCN): c.1538 + 14 T > G) in the intron 13 of the *FLCN* gene (chr17:17214971 (GRCh38.p12) (Fig. 4). It is 14 nucleotides into intron 13, and is considered benign/likely benign by ClinVar (rs112111994, Variation ID: 167073). No variants were found in the other nine exons of the gene studied.

## Discussion and conclusions

The cutaneous manifestations of the present case presented as multiple normochromic papules on the frontal region of the face and on the back, identified as trichodiscomas and consistent with the literature findings [2, 3, 6, 8]. Trichodiscomas and fibrofolliculomas are considered the same type of lesion [6, 21] but represent different evolutionary stages of a single lesion. This idea is reinforced by the fact that these dermatopathies are immunophenotypically similar and thus derived from the same histogenic precursor [21, 22]. In addition, artificial differences in the histological interpretation of these benign tumors may occur due to different sectioning planes of the specimen during histological examination [21].

Pulmonary involvement, which is often the earliest phenotypic manifestation and one of the most frequent in BHDS, was not present in the reported case. Approximately 30% of BHDS patients report a history of spontaneous pneumothorax, usually before the age of 40 years [4]. The patient had no clinical manifestation or image related to pulmonary cysts and/or pneumothorax. Even so, the absence of symptoms or alterations in chest imaging tests does not negate the diagnosis, since pulmonary manifestations may not exist in up to 20% of individuals with BHDS [3–6]. The pathogenic variants of BHDS in exon 9 are associated with the finding of a greater number of pulmonary cysts and the size of these cysts [3–6, 8].

Patients with BHD have a 7-fold increased risk of developing renal cancer [23]. Renal tumors in BHDS occur more frequently in men, and in the fifth and sixth decades of life, they are often bilateral and multifocal with slow growth and are usually asymptomatic in the early stages [3, 6, 7, 9, 24], as was the case with the patient in the present study. In the magnetic resonance angiography, solid renal masses presented images suggestive of an angiomyolipoma, which is a tumour that is reported in the literature to be associated with BHDS [22, 25]. A biopsy of the renal masses was ruled out due to the risk of complications and comorbidities present in the patient. Angiomyolipoma is one of the most common benign solid tumors in the kidney [26] and it is often comorbid with Tuberous sclerosis complex (TSC) [27], a rare autosomal dominant multisystem disorder characterized by benign tumors presenting preferentially in the skin, brain, and kidneys. The clinical picture of TSC is very broad and can range from mild symptoms that do not limit the individual to manifestations with severe disabilities in multiple organ systems, often involving intellectual impairment. TSC is caused by mutations in the *TSC1* or *TSC2* gene [28]. It has been suggested that the BHD and TSC proteins may function within a common pathway. *FLCN* and *TSC1/TSC2* genes regulate common downstream targets, suggesting the existence of an unusual mechanism in which both inappropriate mTOR inhibition in BHD and inappropriate mTOR activation in TSC may lead to overlapping clinical symptoms [23, 29–31]. Skin hamartomas, lung cysts, pneumothorax, and renal tumors also occur in TSC [23].

In the present report, the finding of hyperplastic polyposis involving the stomach, duodenum, jejunum, and colon was observed in the upper digestive endoscopy, enteroscopy, and colonoscopy and was confirmed by the histopathological examination of the biopsies of polyps at these sites. The most frequent types of gastric polyps are represented by hyperplastic polyps, which are related to *H. pylori* infection, as occurred with the patient in this report, and the polyps of the fundic gland that are associated with the use of the proton pump inhibitor. *H. pylori* eradication may induce regression of hyperplastic polyps related to the reduction or even disappearance of inflammation of the gastric mucosa caused by infection with *H. pylori* [32, 33].

The finding of benign or malignant colorectal tumors does not constitute a frequent phenotypic manifestation of BHD syndrome, and there are no reports of colorectal hyperplastic polyps or types of polyps in the other sites of the gastrointestinal tract [34]. In another study [20], the risk of the development of colon neoplasia evaluated in 149 patients with BHDS was 20%, compared with 4.9% in the general population. Differences in environmental exposures or genetic modifiers may have contributed to these divergent results, also these findings raise the possibility that different allelic variants of *FLCN* may cause a predisposition for a greater or lesser risk of colorectal polyps and/or colorectal neoplasia in BHDS [6].

Somatic insertion/deletion variants of *FLCN* in the C8 region of exon 11 have also been reported in 16 to 23% of sporadic colorectal tumors with microsatellite instability [20]. The analysis of genotype-phenotype correlations for two recurrent frameshift variants in *FLCN* identified in families with BHDS demonstrated a significantly increased risk of colorectal neoplasia in carriers of the variant c.1285dupC (within the exon 11 C_8_ mononucleotide tract) than in carriers of the c. 610delGCinsTA pathogenic variant [20]. Nahorski et al. [20] suggested that somatic pathogenic variants of *FLCN* in patients with colorectal cancer are of the “transient” type and are not mutagenic drivers.

In the present report, a germline variant in *FLCN* was found in heterozygosis that corresponded to a single base exchange of timine for guanine, 14 nucleotides into intron 13. The variant found is considered benign/likely benign by ClinVar and may not contribute to the patient’s phenotype. Three records at the ClinVar (RCV000153244.3; RCV000269445.1; RCV000324507.1) associated this variant at *FLCN* gene to spontaneous pneumothorax and multiple fibrofolliculomas.

The clinical history of the patient in question did not indicate the existence of relatives with similar manifestations, but his pathological history provided other relevant information. The patient had conditions that were also described in patients with BHDS: vocal fold angioma, lipomas, thyroid cancer, oncocytoma, and parotid adenoma, as well as basal cell cutaneous carcinoma [4, 6, 8, 24].

On the other hand, the finding of gastrointestinal hyperplastic polyposis could be related to genetic variant in genes such as *STK11*, associated with Peutz-Jeghers syndrome [35]; *APC*, associated with familial adenomatous polyposis [36]; and *PTEN*, associated to Cowden syndrome [37]; or less often in *TSC1/2* genes, associated to Tuberous sclerosis complex, whose gastrointestinal manifestations are uncommon [38]. The search for variants in other genes was not possible in the patient of the present report, due to his death and not autopsy authorization.

Although hyperplastic polyps are the most common benign polyps in the large intestine (particularly in the rectum) and the stomach, they have rarely been described in other parts of the gastrointestinal tract [12, 13, 17, 18], and to the best of our knowledge, the finding of hyperplastic polyps in the jejunum has not yet been described. As the hyperplastic polyps of the colon and, to a lesser degree, the stomach may carry malignant conversion potential, investigating their presence in individuals with BHDS is important.

*FLCN* is currently the only gene known to be associated with BHD; however, the sensitivity of mutation detection is not 100%. In addition to the aforementioned mutation types, intragenic deletions and duplications are also catalogued in the Leiden Open Variation Database (https://databases.lovd.nl/shared/genes/FLCN) [39–41]. Thus, DNA-based diagnosis should ideally consist of sequence analysis and a test for large deletions or duplications, such as multiplex ligation-dependent probe amplification assays (MLPA) [42]. Since clinical expression of BHD varies greatly, BHD should be differentiated from other syndromes with similar signs and symptoms, such as tuberous sclerosis complex, multiple endocrine neoplasia type 1, Cowden syndrome, multiple trichoepitheliomas, basaloid follicular hamartoma syndrome, and generalized hair follicle hamartoma [23]. The diagnosis of BHD must be made based on the combination of clinical examination, skin biopsy, and genetic testing.

## Data Availability

The datasets generated and analysed during the current study are not publicly available because it is possible that individual privacy could be compromised but are available upon reasonable request according to Brazilian Ministry of Health (Ministério da Saúde, Brazil, Conselho Nacional de Saúde, Resolução CNS 340/04). To request the datasets, please contact the corresponding author (bianca.bianco@fmabc.br or bianca.bianco@hotmail.com).

## Abbreviations

AMPK: AMP-activated protein kinase
BHDS: Birt-Hogg-Dubé syndrome
CT: Computed tomography
*FLCN*: Folliculin gene
FLCN: Protein folliculin
HIF: Hypoxia-inducible factors
MRI: Magnetic resonance imaging
MSI: Microsatellite instability
mTOR: Mammalian target of rapamycin signalling pathways
PCR: Polymerase Chain Reaction
TGF-β: Transforming growth factor beta
